# Extending the spectrum of fully integrated photonics to submicrometre wavelengths

**DOI:** 10.1038/s41586-022-05119-9

**Published:** 2022-09-28

**Authors:** Minh A. Tran, Chong Zhang, Theodore J. Morin, Lin Chang, Sabyasachi Barik, Zhiquan Yuan, Woonghee Lee, Glenn Kim, Aditya Malik, Zeyu Zhang, Joel Guo, Heming Wang, Boqiang Shen, Lue Wu, Kerry Vahala, John E. Bowers, Hyundai Park, Tin Komljenovic

**Affiliations:** 1 Nexus Photonics, Goleta, CA USA; 2 Department of Electrical and Computer Engineering, University of California, Santa Barbara, CA USA; 3 T. J. Watson Laboratory of Applied Physics, California Institute of Technology, Pasadena, CA USA

**Keywords:** Integrated optics, Silicon photonics, Semiconductor lasers

## Abstract

Integrated photonics has profoundly affected a wide range of technologies underpinning modern society^1–4^. The ability to fabricate a complete optical system on a chip offers unrivalled scalability, weight, cost and power efficiency^5,6^. Over the last decade, the progression from pure III–V materials platforms to silicon photonics has significantly broadened the scope of integrated photonics, by combining integrated lasers with the high-volume, advanced fabrication capabilities of the commercial electronics industry^7,8^. Yet, despite remarkable manufacturing advantages, reliance on silicon-based waveguides currently limits the spectral window available to photonic integrated circuits (PICs). Here, we present a new generation of integrated photonics by directly uniting III–V materials with silicon nitride waveguides on Si wafers. Using this technology, we present a fully integrated PIC at photon energies greater than the bandgap of silicon, demonstrating essential photonic building blocks, including lasers, amplifiers, photodetectors, modulators and passives, all operating at submicrometre wavelengths. Using this platform, we achieve unprecedented coherence and tunability in an integrated laser at short wavelength. Furthermore, by making use of this higher photon energy, we demonstrate superb high-temperature performance and kHz-level fundamental linewidths at elevated temperatures. Given the many potential applications at short wavelengths, the success of this integration strategy unlocks a broad range of new integrated photonics applications.

## Abstract (web summary)

Fully integrated photonics at submicrometre wavelengths is realized by a heterogeneous integration technology.

## Main

**Fig. 1:**
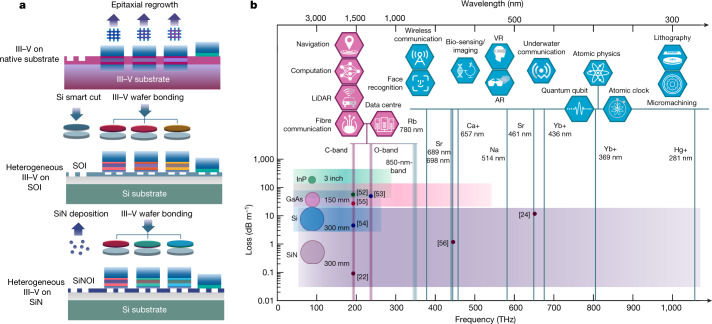
Fully integrated photonic platforms. **a**, The evolution of fully integrated photonic platforms: pure III–V platform relies on multiple epitaxial regrowths to combine active and passives structures; heterogeneous III–V on SOI requires two bonding procedures, the ‘smart-cut’ method to produce an integrated Si film and III–V bonding to transfer III–V epitaxy layers from native substrate onto SOI; the heterogeneous III–V on SiN platform needs only SiN direct deposition to integrate SiN film, and only one wafer bonding process to add the III–V layer. **b**, The spectral coverage of fully integrated PICs: boxes represent the transparency window of passive platforms on the basis of different materials (InP^52^, GaAs^53^, Si^54,55^, SiN^22,24,56^) that can be used for fully integrated PICs, points represent the current state-of-the-art loss on these passive waveguides and wafer marker sizes represent the current maximum wafer scale in foundries. The icons on the upper side represent applications of fully integrated PICs over the spectrum map. Purple icons indicate applications accessible to both existing fully integrated PICs and the III–V/SiN platform of this article; blue icons correspond to applications made possible by the heterogeneous III–V/SiN platform.

Integrated photonics has made rapid progress in the last two decades, and the most crucial steps in its advance have been the emergence of novel integration platforms (Fig. 1a). The earliest photonic integration was based purely on III–V materials on native substrates^9^, in which active and passive photonic components were combined on a chip to form optical systems. This approach led to the first generation of commercially viable photonic technologies. Since then, integrated photonics has benefited from the expansion of the electronics industry, resulting in high-volume adoption of silicon photonics (SiPh). Whereas III–V manufacturing has not grown apace with silicon, it is possible to manufacture photonic integrated circuits (PICs) on large-scale silicon-on-insulator (SOI) wafers by heterogeneously bonding III–V epitaxy in a variety of different ways^10^. Leveraging mature complementary metal–oxide–semiconductor foundry infrastructures, the SOI integrated photonics platform significantly reduces the cost of photonic chips at scale.

Another key factor driving the evolution of integrated photonics is low propagation loss. As SOI waveguides exhibit propagation losses an order of magnitude lower than III–V waveguides^6^, SiPh PICs can accommodate more individual components and thus support more complex photonic systems. Moreover, lower loss boosts the performance of passive structures and coherent light sources. These advantages have driven explosive growth in SiPh, opening up a plethora of new applications, from data centres^6^, to neural networks^11^, to Lidar^12^ and to quantum photonics^13^.

With this broadening of the application scope, however, the limitations of the SOI platform are beginning to surface. One comes from the bandgap wavelength of silicon, of around 1.1 μm (Fig. 1b). Below this wavelength SOI waveguides become highly absorptive. Therefore, ultraviolet (UV), visible and a substantial portion of the near-infrared (near-IR) are currently inaccessible to state-of-the-art integrated photonics. This restriction prohibits on-chip solutions in important fields, such as atomic physics, augmented reality/virtual reality, biosensing and quantum communications^14–20^, as shown in Fig. 1b.

One promising path towards addressing this problem is to implement passive structures using silicon nitride (SiN)^21^, whose waveguides exhibit extremely low losses of less than 0.1 dB m^−1^ at telecommunication wavelengths^22,23^ and remain scattering-limited down to below 460 nm (ref. ^24^), making them attractive for ultra-high-Q microcavities, narrow linewidth lasers and non-linear devices, such as microcomb sources and on-chip frequency converters. Furthermore, because SiN wafers are produced by direct deposition on a Si substrate, they do not require any expensive smart-cut process, suggesting an opportunity to further reduce the cost of foundry-manufactured PICs.

However, until recently, the integration of active components onto SiN PICs has been impeded by the large index mismatch between SiN (approximately 2) and III–V materials (>3). SiN and III–V structures have been integrated on the same substrate to form highly coherent lasers and microcombs at telecommunication wavelengths, but only with an intermediary Si layer for passive–active transitions, which still prohibits short-wavelength operation^25,26^.

This work presents a new generation of integrated photonics with active and passive elements united in a heterogeneously integrated III–V/SiN platform. This integration scheme offers a fully integrated submicrometre photonics platform with versatile building blocks, including lasers, semiconductor optical amplifiers (SOAs), modulators, photodetectors and various passive elements. The combination of a III–V gain section with SiN external cavities yields a heterogeneously integrated, narrow linewidth, widely tunable laser operating beyond the bandgap energy of Si, a device with tremendous implications for atomic physics, sensing and precise metrology. Moreover, the short-wavelength platform exhibits superior high-temperature performance among coherent light sources, which can be used to improve power efficiency in data centres and other hot environments. These results herald the mass production of PICs covering a much broader spectrum and open doors to many new applications.

## Heterogeneously integrated III–V on SiN photonics platform

**Fig. 2:**
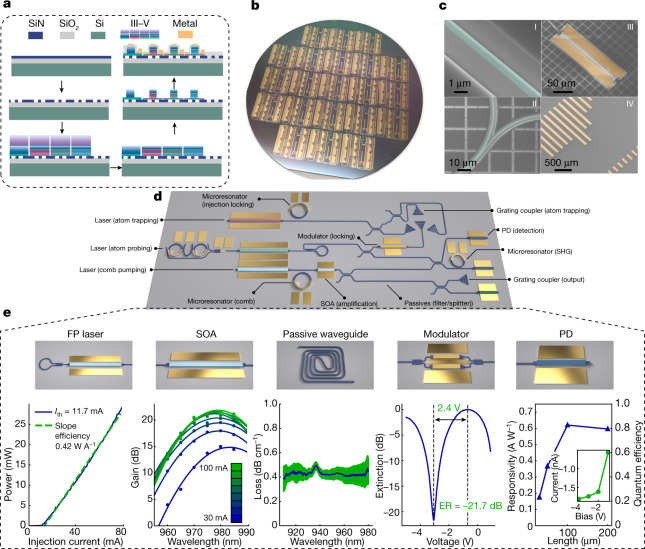
Silicon nitride heterogeneous photonics platform with a full set of passive and active building block components supporting submicrometre wavelengths. **a**, Simplified wafer-scale process flow. Steps shown: (1) SiN deposition on a thermally oxidized Si substrate; (2) SiN waveguide patterning; (3) bonding of multiple III–V epitaxial structures; (4) substrate removal of the III–V epitaxy; (5) III–V processing, including multiple dry/wet etches to form the p–n junctions for active devices; (6) dielectric cladding deposition, via etches and metallization that complete the device fabrication. **b**, A photograph of a fully processed 4 inch wafer that contains thousands of devices. **c**, Scanning electron microscope images of (I) a SiN waveguide, (II) a waveguide coupler, (III) a III–V waveguide and electrical contacts for active components and (IV) an array of lasers and photodiodes connected by SiN waveguides. **d**, Envisioned schematic of a fully integrated atomic clock system fabricated on a single chip. **e**, Active and passive functionalities supported on the platform, with characteristic performance. Left to right: FP laser, a Fabry–Perot laser with integrated broadband mirrors that has less than 12 mA current threshold and more than 25 mW output power to a SiN waveguide; SOA, a semiconductor optical amplifier with maximum gain of 22 dB at 980 nm with 100 mA bias current (the 3 dB bandwidth of the gain at 100 mA spans over 20 nm); passive waveguide, SiN waveguides with sub-dB cm^−1^ propagation loss in the 900–980 nm wavelength range (green shading indicates the standard error from cutback loss linear fit after averaging device loss over identical test structures from a single wafer); modulator, a Mach–Zehnder interferometer with phase modulators showing *V*_π_ = 2.4 V and a greater than 20 dB extinction ratio (ER); PD, a photodiode with a greater than 0.6 A W^−1^ responsivity at 980 nm and nA-level dark current.

Heterogeneous III–V/SiN photonic devices consist of III–V-based epitaxial layer structures bonded on top of SiN waveguides. A simplified fabrication process flow for the III–V/SiN heterogeneous photonic devices is illustrated in Fig. 2a, with a detailed description provided in the Methods. Figure 2b shows a photograph of a completed wafer with hundreds of lasers fabricated on a 4 inch silicon substrate. Scanning electron microscope images (Fig. 2c (I–IV)) show a single SiN waveguide, a coupler, a III–V waveguide with III–V/SiN coupler on one side and an array of lasers connecting with an array of photodiodes via SiN waveguides, respectively.

An essential feature of the platform is efficient light coupling between III–V and SiN waveguides. The large refractive index of III–V material compared to SiN leads to a highly localized optical mode in the III–V layer for a III–V/SiN heterogeneous waveguide. This is a fundamental distinction from a typical III–V/Si heterogeneous waveguide, in which the similar refractive indices of Si and III–V make it possible for the optical mode to hybridize in both materials^27^. As a result, the usual adiabatic coupling scheme based on evanescent fields, although well suited to III–V/Si photonics, does not serve well for III–V/SiN. Butt coupling, a non-adiabatic method widely used in conventional optics, is advantageous in this case. However, efficient butt coupling requires maximal spatial overlap between the waveguides being coupled, which is unobtainable in a wafer-scale heterogeneous integration platform because the bonded layers cannot be vertically aligned. The following III–V/SiN coupler structure addresses this challenge by combining both aforementioned coupling schemes: an intermediary waveguide is patterned in the dielectric cladding between the III–V and SiN waveguides; at the III–V end, the geometry of the intermediary waveguide is optimized for butt coupling; and on the SiN end, it is optimized for adiabatic evanescent coupling to the SiN waveguide. A coupling efficiency of up to 70% was demonstrated in the first generation, and 90% efficiency is achievable with optimal design^28^. Additional details are described in the Supplementary Information.

Figure 2d, showing a proposed integrated PIC for an integrated atomic clock system, illustrates the potential of a fully integrated, short-wavelength PIC ecosystem with direct III–V/SiN coupling. The essential components have been implemented and characterized around 980 nm, as shown in Fig. 2e. Fabry–Perot (FP) lasers, formed with near-100% loop mirrors on the back side and 10% mirrors on the front side, provide a light source. An 800 μm long FP laser exhibits a low threshold current of 12 mA, whereas the output power and slope efficiency exceed 25 mW and 0.38 W A^−1^, respectively. Integrated SOAs are fabricated with more than 22 dB optical gain and 20 nm 3 dB bandwidth. For detection, III–V photodiodes (PDs) exhibit nA-level dark current and more than 0.6 A W^−1^ responsivity and 80% quantum efficiency at 980 nm. We also demonstrate a 2 mm long phase shifter using the same GaAs epitaxial material with a *V*_π_ of only 2.4 V and Mach–Zehnder modulators with more than 22 dB extinction ratio, measured at a wavelength of 1,060 nm. Complementing the III–V active elements are SiN passive waveguides, with loss reaching below 0.5 dB cm^−1^ measured near 980 nm, which corresponds to a quality factor (Q) above 1.5 × 10^6^.

It is also worth noting that recently developed ultra-low-loss SiN waveguides^24,29^ can further reduce waveguide loss by two orders of magnitude. This thin SiN platform will have a greater effective index mismatch between passive and active waveguides, but efficient coupling can still be achieved with the same coupling strategy.

## Integrated coherent laser beyond silicon bandgap

One key application of heterogeneous photonics is coherent lasing. At the telecommunication band, for example, low-loss silicon waveguides have been paired with InP-based optical gain material to produce integrated narrow linewidth lasers^30^. By uniting high-quality SiN passives with short-wavelength III–V gain, our platform offers a similar capability beyond the silicon bandgap limit.

**Fig. 3:**
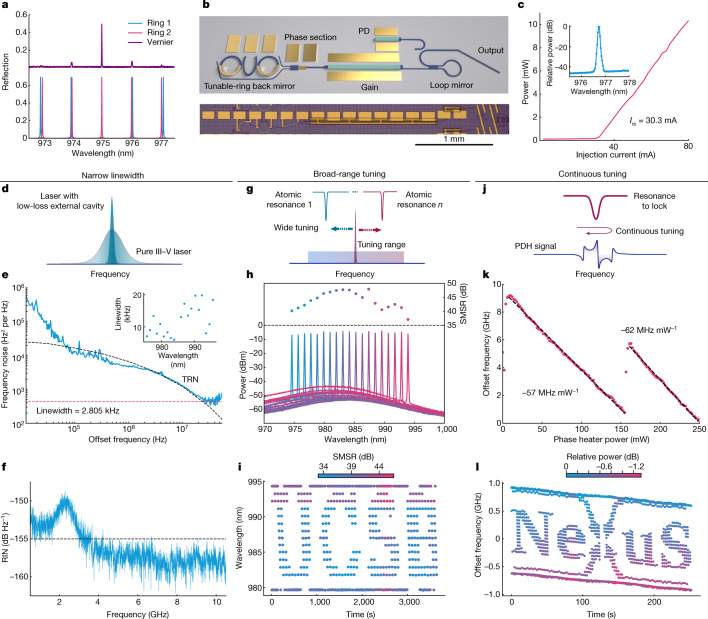
Integrated coherent, widely tunable lasers on silicon nitride. **a**, The wavelength response of individual ring resonators and the resulting measured Vernier spectrum with two rings of different free spectral range. **b**, Schematic of a dual-ring tunable laser with a back mirror formed by two ring resonators in a 100% loop mirror, a 50% reflectivity front loop mirror and a GaAs-based SOA section in between. Thermal microheaters are fabricated on the rings and a part of the laser cavity to align the rings, select the wavelength and tune the round-trip phase accumulation. The photograph shows a tunable laser chip with a form factor of less than 3 × 0.3 mm^2^. **c**, LI characteristic of the laser at a fixed wavelength, showing 30.3 mA threshold current and more than 10 mW output power. Inset: single-mode lasing spectrum. **d**, Improved linewidth with low-loss SiN external cavity. **e**, Frequency noise spectrum, simulated thermorefractive noise and a white noise floor of 450 Hz^2^/Hz, corresponding to a 2.8 kHz Lorentzian linewidth (2π times the white noise floor). Inset: Lorentzian linewidth at 25 °C across the laser tuning range. **f**, Relative intensity noise (RIN), less than −155 dB Hz^−1^ outside the relaxation oscillation resonance. **g**, Wide tuning range enables access to many atomic resonances. **h**, Vernier wavelength tuning of more than 20 nm wavelength with high SMSRs across the whole range. **i**, A ‘UCSB’ logo created by stepping the wavelength of the laser over time. The colour of each dot indicates the measured SMSR at that time step. **j**, Mechanism of locking a resonance with a single continuous tuning parameter, crucial for locking to atomic transitions. **k**, Mode-hop-free, continuous tuning of the III–V/SiN laser frequency obtained over more than 8 GHz by sweeping the phase-tuning section alone. **l**, A ‘Nexus’ logo created by tuning the laser frequency without mode hop, showing great stability and precise control over time.

An integrated laser operating at 980 nm, which consists of a GaAs gain region and a SiN external cavity, is presented as a proof of concept. Figure 3a,b shows the principle of Vernier rings and the schematic design of the laser, whose details are provided in the Methods. The output power from the laser is greater than 10 mW near the gain peak, as shown in the LI (light–current) curve in Fig. 3c, where the power is measured while wavelength is maintained around 976.5 nm. For a fixed gain current of 75 mA, the power output is measured to be higher than 6 mW across the whole wavelength range.

A compact laser, with a footprint of less than 1 mm^2^, as shown in Fig. 3b, is valuable for a wide range of applications at short wavelengths^31^. One important example is in atomic physics. The III–V/SiN heterogeneous laser described here offers a performance comparable to a bulky external cavity-diode laser^32,33^, but with the form factor of a fully integrated device. Figure 3e shows the two-sided power spectral density of the laser noise at 980 nm wavelength, measured with a delayed self-heterodyne setup and cross-correlation technique (Methods). The spectrum is dominated by 1/*f* noise at low-offset frequency (*f*) range, as commonly observed in semiconductor lasers. Between 100 kHz to 30 MHz the laser noise is mainly dominated by thermorefractive noise (Methods). At around 30 MHz offset frequency, a white noise floor of 450 Hz^2^/Hz is reached, corresponding to a Lorentzian linewidth of 2.8 kHz, with a 10 kHz-level linewidth across the whole tuning range (Fig. 3e). Unlike previous integrated pure III–V lasers, whose fundamental linewidths (typically above 100 kHz (ref. ^6^)) are broader than many atomic transition lines^34,35^, the III–V/SiN heterogeneous laser presented here derives significant noise reduction from its low-loss SiN ring-resonator-based mirror, opening access to those narrow-line atomic transitions. The III–V/SiN heterogeneous laser also shows good amplitude noise performance, with relative intensity noise lower than −155 dB Hz^−1^ (noise floor of the measurement tool) outside the relaxation oscillation resonance near the 2 GHz offset frequency, as shown in Fig. 3f.

Another key feature of the Vernier laser design is its wide tunability. With only narrow tuning capability, producing specific wavelengths (for example, targeting atomic transitions) demands tight fabrication tolerances. Using microheaters placed on top of the ring resonators, one can make use of the thermo-optic effect to tune each ring comb, shifting the Vernier location to the desired wavelengths. This simple Vernier comb principle provides a mechanism to obtain a reconfigurable optical filter on-chip, which is key to a widely tunable laser. Figure 3h shows the lasing spectra measured by coarsely stepping the wavelength in 1 nm increments, characterized at 25 °C. The tuning range is about 20 nm (equivalent to approximately 6 THz), primarily limited by the gain bandwidth from the 980 quantum wells. The lasing side-mode-suppression ratio (SMSR) is greater than 35 dB across the entire tuning range, and approaches 50 dB when the lasing wavelength is located near the gain peak, as shown in the figure inset. The wavelength of the laser can be stepped repeatably over a wide range without sacrificing SMSR, as shown in Fig. 3i, in which the *y* axis shows the lasing wavelength as a function of time and the dot colour indicates the SMSR of the lasing mode.

In addition to broad tuning, when locking a laser to a high-Q cavity or atomic transitions, continuous fine tuning is often required over a smaller range. As shown in Fig. 3k, by simply sweeping the phase tuner, our laser supports a mode-hop-free tuning range of 8 GHz. Note that a much larger mode-hop-free tuning range can be achieved by simultaneously tuning the rings and the phase section^36^. As shown in Fig. 3l, frequency can also be repeatably and precisely controlled over several GHz.

## High-temperature advantage of short-wavelength PICs

**Fig. 4:**
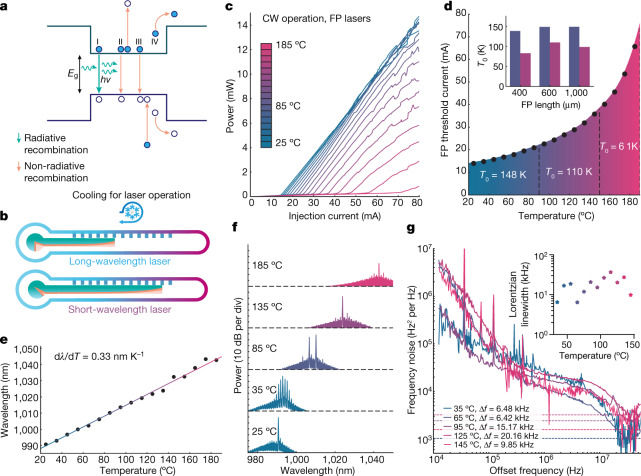
Extremely high temperature, fully integrated lasers. **a**, Simplified band diagram of laser and illustration of major carrier recombination and leakage processes, including (I) radiative recombination, (II) Auger recombination, (III) intervalence band absorption and (IV) carrier leakage over the hetero-barriers. **b**, The temperature dependence of carrier recombination processes for fully integrated long-wavelength lasers and the short-wavelength lasers in this work. Non-radiative recombination increases exponentially with temperature, but the effect is reduced in a short-wavelength GaAs platform due to the increased energy bandgap and quantum well depth. The allowed working temperature is represented by the length of the solid bar, and a cooling process is necessary if the free-running temperature of the device goes beyond the working temperature range. **c**, LI characteristics of SiN heterogeneous FP lasers from 25 °C to 185 °C. **d**, Threshold current of the laser versus temperature, extracted from the LI curves. Characteristic temperature *T*_0_ = 148 K within 20 °C–90 °C; *T*_0_ = 110 K in the range from 90 °C to 150 °C, the highest lasing temperature reported in a commercial heterogeneously integrated laser^1^, and *T*_0_ = 61 K above 150 °C. Inset: characteristic temperatures, *T*_0_, in the first two ranges (20 °C–90 °C and 90 °C–150 °C) of FP lasers with various cavity length. **e**, FP lasing wavelength versus temperature, illustrating linear gain red shift of 0.33 nm K^−1^. **f**, FP lasing spectra at select temperatures. **g**, Select frequency noise spectra of the single-mode tunable laser measured at elevated temperatures, showing less than 10 kHz Lorentzian linewidth even at 145 °C. Inset: fundamental linewidth versus temperature from 35 °C to 145 °C.

A major challenge for integrated photonics is the requirement of active cooling. As the performance of diode lasers degrades at elevated temperatures, it is necessary to cool the PICs to maintain performance. Thermal degradation of lasers is caused by gain reduction due to the wider spreading of the Fermi distribution of carriers at increased temperature^37^ and by the loss of radiative carriers via various mechanisms, notably including carrier leakage over hetero-barriers^38^, Auger recombination^38,39^ and intervalence band absorption^40,41^ (Fig. 4a), all of which exponentially increase with temperature. Of these three carrier-loss mechanisms, Auger recombination and intervalence band recombination both decrease exponentially with material bandgap^38,41^. Hence, shorter wavelength lasers are inherently more resilient to these non-radiative loss processes. In addition, the material systems grown on GaAs substrates used for near-IR- to visible-wavelength lasers have a favourably larger conduction band offset than that of the InP system of longer wavelengths, and thus higher quantum well barriers and better carrier confinement at elevated temperature^38^. Together, the above effects give the short-wavelength GaAs platform superior high-temperature performance (Fig. 4b), which could significantly reduce power consumption by operating with only passive cooling.

To study thermal performance, our heterogeneous III–V/SiN FP lasers were characterized by LI measurements at stage temperatures from 25 °C up to 185 °C, as shown in Fig. 4c. Continuous-wave lasing was achieved up to 185 °C, which is the highest operation temperature among all lasers integrated on a silicon chip so far, and significantly higher than the previous record (150 °C)^1^. Threshold currents up to 90 °C are well described by an exponential model with a characteristic *T*_0_ of 148 K (Fig. 4d), which is on a par with the best thermal performance among diode lasers on native substrate^42^. Additionally, spectral measurements indicate red-shifting of the lasing wavelength window at a rate of 0.33 nm K^−1^, with a maximum lasing wavelength of 1,044.5 nm at 185 °C, which is more than 50 nm redder than at room temperature, as shown in Fig. 4e,f.

Beyond simply lasing, the III–V/SiN heterogeneous platform also demonstrates integrated narrow linewidth lasers at elevated temperature, showing great promise for applications, including coherent communications in data centres, remote sensing or metrology in harsh environments. Ring-resonator-based tunable lasers (similar to those in the previous section) were characterized. Phase noise measurements were carried out at temperatures from 35 °C up to 145 °C (Methods). The best overall fundamental linewidth measured was lower than 7 kHz, and a linewidth of lower than 10 kHz was measured at 145 °C. Only minimal linewidth degradation was observed (Fig. 4e). Note that integrating both III–V and SiN on the same substrate ensures a robust coupling between the gain and the external cavity over a broad temperature range, whereas other linewidth-narrowing methods, such as hybrid integration with chip-to-chip butt coupling^23,29,43^, face positional misalignment challenges due to thermal expansion mismatch between different elements.

## Discussion

Using the integration strategy demonstrated in this work, the wavelength range of silicon photonics can be extended down to green wavelengths with GaAs-based material (GaP, InGaP, AlGaAs), and down into blue, violet and UV ranges by incorporating GaN-based material. With the ultra-low-loss SiN waveguide recently characterized at blue and violet wavelengths^24^, it will be possible to produce scalable PICs throughout the entire visible wavelength range. By using high-Q SiN cavities, fully integrated non-linear systems may also be realized on this platform, such as microcombs^44–46^, stimulated Brillouin lasers^47^ and strong frequency conversion systems^48^. The same integration strategy is well suited to different thicknesses of SiN waveguide, including thinner ones (<100 nm) for ultra-high-Q or thick SiN (>700 nm) for microcomb generation in the anomalous dispersion regime. Other materials, such as LiNbO_3_, AlN, SiC, AlGaAs and chalcogenide glass, can also be used intermittently as the media for passive waveguides, further enriching the toolbox of integrated photonics and extending the spectrum of PICs towards longer wavelengths (>10 μm) not supported by current PICs.

Short-wavelength PICs have the potential to rewrite the map of photonics applications. In atomic physics, short-wavelength PICs will support on-chip atomic clocks and quantum computing with trapped ion qubits^14^. With a platform spanning the vast wavelength range from visible to telecommunication, coherent links can be designed to support octave-spanning self-reference systems for time–frequency metrology^49^ and visible-telecommunication entanglement in quantum communication^50^. In the consumer market, improved high-temperature performance will relax the cooling requirements of photonic devices, providing an energy-efficient solution for data centres and photonic computation. By combining highly coherent light sources at visible ranges with low-loss optical phase arrays^51^, the III–V/SiN heterogeneous photonics platform can potentially remove bulky lens imaging systems from augmented reality/virtual reality equipment, making it lighter and more power efficient.

Finally, because the fabrication of this platform is compatible with existing photonic foundries producing heterogeneous III–V/Si photonics, we expect that this technology will soon be adopted for larger scale high-volume production. As the material cost of SiN-on-insulator is lower than that of SOI, this development will make III–V/SiN economically preferable to the now-ubiquitous III–V/Si, reducing costs throughout the industry and truly revolutionizing integrated photonics.

## Methods

### Device fabrication

A silicon substrate was thermally oxidized to form a suitable SiO_2_ base layer for waveguide cladding. Next, a 350 nm thick SiN film was deposited by low-pressure chemical vapour deposition, which was then patterned with a photolithographic stepper system and dry etched to form passive waveguide structures. The III–V epitaxy was directly bonded onto the nitride wafers in an optimized molecular bonding process. As the III–V is not patterned beforehand, no precise alignment was needed for the bonding, enabling wafer-scale, high-volume production, which is one key advantage of heterogeneous integration over other integration strategies, such as chip-to-chip packaging or transfer printing. In this work, the III–V epitaxy was a GaAs/AlGaAs-based layered structure with InGaAs/GaAsP quantum wells. Although GaAs/AlGaAs materials with quantum dots have been heterogeneously integrated onto SOI waveguides previously^57^, GaAs/AlGaAs quantum wells are heterogeneously integrated here for the first time. The detailed layer structure can be found in the Supplementary Information. To enhance the bonding strength between GaAs/AlGaAs and SiN, we deposited a 7 nm Al_2_O_3_ layer on the epi surface as an adhesion layer before bonding. Also, to manage the large thermal expansion coefficient difference between GaAs and Si, the post-bond anneal was performed at low temperature (150 °C) but with a long duration (up to 12 h) to enhance the bonding strength. The III–V substrates were then removed by mechanical polishing and a selective wet etch before continuing the III–V process to form active components. A blanket dielectric was deposited to form the top cladding for both SiN and III–V waveguides, as well as the insulator between metal sections. Finally, vias were opened and metal pads deposited to form electrical contacts to the devices. A more detailed description of the fabrication process can be found in the Supplementary Information.

### Tunable laser design

Figure 3b shows the schematic design of the laser, whose back mirror consists of two ring resonators cascaded within a loop in an add-drop configuration. Each ring resonator forms a comb in the wavelength domain, with adjacent comb lines separated by one free spectral range. As shown in Fig. 3a, by choosing radii for the rings so that the free spectral ranges of the two combs are slightly different, the dual-ring mirrors reflective spectrum, which is the product of these two combs, is a Vernier comb that has only a single dominant comb line at which the two individual comb lines align. In addition to the Vernier tunable ring mirror, the laser also features a phase-tuning element and an on-chip monitor photodiode.

### Noise characterization

The delayed self-heterodyne phase noise measurements were performed using a 1 km delay line and a Brimrose acousto-optic modulator (TEM-110-10-55-980-2FP) on opposing arms of a Mach–Zehnder interferometer. The two output signals were sent to Newport low noise photoreceivers (model 1801) and recorded with a Tektronix 5 Series mixed signal oscilloscope for cross-correlation analysis as in ref. ^58^. During the measurement, all laser inputs and the thermo-electric cooler of the measurement stage (see below) were controlled by Lightwave ILX LDX-3620B ultra-low-noise battery current sources.

### Thermorefractive noise simulation

Here, we derive the thermorefractive noise of the integrated laser resonator based on the fluctuation–dissipation theorem^59^ and simulate the thermorefractive noise of the dual-ring tunable laser in Fig. 3 with a COMSOL multiphysics finite element method solver. For a single optical mode within the resonator, by solving the Helmholtz equation, the optical resonance angular frequency *ω*_opt_ can be expressed as

1 $${\omega }_{{\rm{o}}{\rm{p}}{\rm{t}}}^{2}={c}^{2}\frac{\int {|{\rm{\nabla }}\times {\bf{\text{E}}}|}^{2}{{\rm{d}}}^{3}{\bf{\text{r}}}}{\int {n}_{0}^{2}{|{\bf{\text{E}}}|}^{2}{{\rm{d}}}^{3}{\bf{\text{r}}}}$$

where **E** is the modal electric field, **r** is the spatial coordinate vector, *c* is the vacuum speed of light and *n*_0_ is the refractive index of the material.

Under thermal shifts, the refractive index change ∆*n* can be expressed as ∆*n* = *n*_0_*β*_*n*_δ*T*, where *β*_*n*_ is the thermo-optic coefficient. The resonant frequency shift, δ*ω*_opt_, can be solved as

2 $$\delta {\omega }_{{\rm{o}}{\rm{p}}{\rm{t}}}=\int \frac{1}{N}{n}_{0}^{2}{\beta }_{n}{\omega }_{{\rm{o}}{\rm{p}}{\rm{t}}}{|{\bf{\text{E}}}|}^{2}\delta T{{\rm{d}}}^{3}{\bf{\text{r}}},$$

where *N* is the normalization for optical mode intensity, *N* = ∫*n*_0_*n*_g_ |*E*|^2^d^3^*r*.

The resonator exists in a heat reservoir with temperature *T*_0_ and the temperature deviation from thermal equilibrium follows the heat equation in the frequency domain:

3 $${\rm{i}}\omega \rho {c}_{{\rm{p}}}\delta T=\kappa {{\rm{\nabla }}}^{2}\delta T-{\rm{i}}\omega {Q}_{{\rm{e}}{\rm{x}}{\rm{t}}}$$

where *ρ* is the material density, *c*_p_ is the heat capacity, *κ* is the thermal conductivity and *Q*_ext_ is the fictitious external heat source, given by

4 $${Q}_{{\rm{e}}{\rm{x}}{\rm{t}}}=\frac{{f}_{0}}{N}{n}_{0}^{2}{\beta }_{n}{\omega }_{{\rm{o}}{\rm{p}}{\rm{t}}}{|{\bf{\text{E}}}|}^{2}{T}_{0}.$$

Here *f*_0_ is a conversion unit to energy.

The resulting dissipated power *W*_diss_ can be calculated as:

5 $${W}_{{\rm{d}}{\rm{i}}{\rm{s}}{\rm{s}}}=\frac{1}{2}\int \kappa \frac{{|{\rm{\nabla }}\delta T|}^{2}}{{T}_{0}}{{\rm{d}}}^{3}{\bf{\text{r}}}$$

According to the fluctuation–dissipation theorem, the two-sided power spectral density (SD) of resonant frequency *ω* can be expressed as

6 $${{\rm{SD}}}_{\omega \omega }=\frac{4{k}_{{\rm{B}}}T}{{\omega }^{2}}\frac{{W}_{{\rm{diss}}}}{{{\rm{| }}{f}_{0}{\rm{| }}}^{2}}$$

Critical parameters used in the simulation of thermal properties are: *ρ*(Si_3_N_4_) = 2.2 × 10^3^ kg m^−3^ (ref. ^60^), *ρ*(SiO_2_) = 2.2 × 10^3^ kg m^−3^, *C*(Si_3_N_4_) = 600 J kg^−1^ K^−1^ (ref. ^60^), *C*(SiO_2_) = 740 J kg^−1^ K^−1^, *κ*(Si_3_N_4_) = 2.23 W m^−1^ K^−1^ (ref. ^61^), *κ*(SiO_2_) = 1.4 W m^−1^ K^−1^, *β*_*n*_(Si_3_N_4_) = 2.4 × 10^−5^ K^−1^, *β*_*n*_(SiO_2_) = 1.0 × 10^−5^ K^−1^ and ambient temperature of 293.15 K.

### High-temperature measurement

The high-temperature measurement stage consisted of four levels: a heater, a heat spreader, a thermo-electric cooler and the measurement stage. Additionally, polyimide tape was applied to reduce heat flow to the air, and a hood-like structure of aluminium was added to shield the device under test from air currents. Temperature was monitored using a Vescent SLICE-QTC controller with EPCOS-TDK thermistors (B57540G1103F005) in the stage and the heat spreader. Output power was measured with a calibrated Newport integrating sphere (819C-UV-5.3-CAL). Lasing wavelength was determined by measuring spectra with a Yokogawa Optical Spectrum Analyzer (AQ6374).

## Online content

Any methods, additional references, Nature Research reporting summaries, source data, extended data, supplementary information, acknowledgements, peer review information; details of author contributions and competing interests; and statements of data and code availability are available at 10.1038/s41586-022-05119-9.

## Supplementary information

Supplementary Information Supplementary Notes 1–4 and Figs. 1–9.

## Data Availability

The data presented in this paper’s figures are available on https://zenodo.org/record/6757842#.YrknDtLMKV4.

## Code availability

The codes that support the findings of this study are available from the corresponding authors upon reasonable request.

## References

[1] Jones R (2019). Heterogeneously integrated InP\/silicon photonics: fabricating fully functional transceivers. IEEE Nanotechnol. Mag..

[2] Doerr C, Chen L, Chen L, Ton D (2021). Linear 2D beam steering with a large focusing grating via focal point movement and wavelength.. IEEE Photon. Technol. Lett..

[3] Wang J (2020). Silicon-based integrated label-free optofluidic biosensors: latest advances and roadmap. Adv. Mater. Technol..

[4] Shen Y (2017). Deep learning with coherent nanophotonic circuits. Nat. Photon..

[5] Heck MJR, Bauters JF, Davenport ML, Spencer DT, Bowers JE (2014). Ultra-low loss waveguide platform and its integration with silicon photonics. Laser Photon. Rev..

[6] Margalit N (2021). Perspective on the future of silicon photonics and electronics. Appl. Phys. Lett..

[7] Atabaki AH (2018). Integrating photonics with silicon nanoelectronics for the next generation of systems on a chip. Nature.

[8] Fathololoumi S (2021). 1.6 Tbps silicon photonics integrated circuit and 800 Gbps photonic engine for switch co-packaging demonstration. J. Lightwave Technol..

[9] Merz JL, Yuan YR, Vawter GA (1985). Photonics for integrated circuits and communications. Opt. Eng..

[10] Komljenovic T (2018). Photonic integrated circuits using heterogeneous integration on silicon. Proc. IEEE.

[11] Zhang Q, Xing Z, Huang D (2021). Implementation of pruned backpropagation neural network based on photonic integrated circuits. Photonics.

[12] Poulton CV (2017). Coherent solid-state LIDAR with silicon photonic optical phased arrays. Opt. Lett..

[13] Qiang X (2018). Large-scale silicon quantum photonics implementing arbitrary two-qubit processing. Nat. Photon..

[14] Mehta KK (2020). Integrated optical multi-ion quantum logic. Nature.

[15] Niffenegger RJ (2020). Integrated multi-wavelength control of an ion qubit. Nature.

[16] Hummon MT (2018). Photonic chip for laser stabilization to an atomic vapor with 10^−11^ instability. Optica.

[17] Masood T, Egger J (2021). Augmented reality: focusing on photonics in industry 4.0. IEEE J. Sel. Top. Quantum Electron..

[18] Zinoviev KE, González-Guerrero AB, Domínguez C, Lechuga LM (2011). Integrated bimodal waveguide interferometric biosensor for label-free analysis. J. Lightwave Technol..

[19] Orieux A, Diamanti E (2016). Recent advances on integrated quantum communications. J. Opt..

[20] Blumenthal DJ (2020). Photonic integration for UV to IR applications. APL Photon..

[21] Krückel CJ, Fülöp A, Ye Z, Andrekson PA, Torres-Company V (2017). Optical bandgap engineering in nonlinear silicon nitride waveguides. Opt. Express.

[22] Puckett MW (2021). 422 Million intrinsic quality factor planar integrated all-waveguide resonator with sub-MHz linewidth. Nat. Commun..

[23] Li B (2021). Reaching fiber-laser coherence in integrated photonics. Opt. Lett..

[24] Morin TJ (2021). CMOS-foundry-based blue and violet photonics. Optica.

[25] Xiang C (2020). Narrow-linewidth III-V/Si/Si_3_N_4_ laser using multilayer heterogeneous integration. Optica.

[26] Op de Beeck C (2020). Heterogeneous III–V on silicon nitride amplifiers and lasers via microtransfer printing. Optica.

[27] Fang AW (2006). Electrically pumped hybrid AlGaInAs-silicon evanescent laser. Opt. Express.

[28] Park H, Zhang C, Tran MA, Komljenovic T (2020). Heterogeneous silicon nitride photonics. Optica.

[29] Jin W (2021). Hertz-linewidth semiconductor lasers using CMOS-ready ultra-high-Q microresonators. Nat. Photon..

[30] Tran MA, Huang D, Bowers JE (2019). Tutorial on narrow linewidth tunable semiconductor lasers using Si/III–V heterogeneous integration. APL Photon..

[31] Wieman CE, Hollberg L (1991). Using diode lasers for atomic physics. Rev. Sci. Instrum..

[32] Arnold AS, Wilson JS, Boshier MG (1998). A simple extended-cavity diode laser. Rev. Sci. Instrum..

[33] Liu K, Littman MG (1981). Novel geometry for single-mode scanning of tunable lasers. Opt. Lett..

[34] Vogel KR, Dinneen TP, Gallagher A, Hall JL (1999). Narrow-line Doppler cooling of strontium to the recoil limit. IEEE Trans. Instrum. Meas..

[35] McFerran JJ, Luiten AN (2010). Fractional frequency instability in the 10^−14^ range with a thermal beam optical frequency reference. J. Opt. Soc. Am. B.

[36] van Rees A (2020). Ring resonator enhanced mode-hop-free wavelength tuning of an integrated extended-cavity laser. Opt. Express.

[37] Piprek J, Abraham P, Bowers JE (2000). Self-consistent analysis of high-temperature effects on strained-layer multiquantum-well InGaAsP-InP lasers. IEEE J. Quantum Electron..

[38] Coldren, L. A., Corzine, S. W. & Mashanovitch, M. L. *Diode Lasers and Photonic Integrated Circuits* (John Wiley & Sons, 2012).

[39] Braithwaite J, Silver M, Wilkinson VA, O’Reilly EP, Adams AR (1995). Role of radiative and nonradiative processes on the temperature sensitivity of strained and unstrained 1.5 μm InGaAs(P) quantum well lasers. Appl. Phys. Lett..

[40] Childs GN, Brand S, Abram RA (1986). Intervalence band absorption in semiconductor laser materials. Semicond. Sci. Technol..

[41] Adams, A. R., O’Reilly, E. P. & Silver, M. in *Semiconductor Lasers I* (ed. Kapon, E.) 123–176 (Academic Press, 1999).

[42] Derry PL (1992). Low threshold current high-temperature operation of InGaAs/AlGaAs strained-quantum-well lasers.. IEEE Photon. Technol. Lett..

[43] Franken CAA (2021). Hybrid-integrated diode laser in the visible spectral range. Opt. Lett..

[44] Shen B (2020). Integrated turnkey soliton microcombs. Nature.

[45] Gaeta AL, Lipson M, Kippenberg TJ (2019). Photonic-chip-based frequency combs. Nat. Photon..

[46] Chang Lin, Liu Songtao, Bowers John E. (2022). Integrated optical frequency comb technologies. Nat. Photon..

[47] Gundavarapu S (2019). Sub-hertz fundamental linewidth photonic integrated Brillouin laser. Nat. Photon..

[48] Lu X, Moille G, Rao A, Westly DA, Srinivasan K (2021). Efficient photoinduced second-harmonic generation in silicon nitride photonics. Nat. Photon..

[49] Kues M (2019). Quantum optical microcombs. Nat. Photon..

[50] Reimer C (2016). Generation of multiphoton entangled quantum states by means of integrated frequency combs. Science.

[51] Poulton CV (2017). Large-scale silicon nitride nanophotonic phased arrays at infrared and visible wavelengths. Opt. Lett..

[52] D’Agostino D (2015). Low-loss passive waveguides in a generic InP foundry process via local diffusion of zinc. Opt. Express.

[53] Ferguson A (2006). Low-loss, single-mode GaAs/AlGaAs waveguides with large core thickness. IEE Proc. Optoelectron..

[54] Biberman A, Shaw MJ, Timurdogan E, Wright JB, Watts MR (2012). Ultralow-loss silicon ring resonators. Opt. Lett..

[55] Bellegarde C (2018). Improvement of sidewall roughness of submicron SOI waveguides by hydrogen plasma and annealing. IEEE Photon. Technol. Lett..

[56] Chauhan N (2021). Visible light photonic integrated Brillouin laser. Nat. Commun..

[57] Wan Y (2021). High speed evanescent quantum-dot lasers on Si. Laser Photon. Rev..

[58] Wang, H., Wu, L., Yuan, Z. & Vahala, K. in *Conference on Lasers and Electro-Optics* (eds. Kang, J. et al.) SF2O. 2 (Optica Publishing Group, 2021).

[59] Levin Y (2008). Fluctuation–dissipation theorem for thermo-refractive noise. Phys. Lett. A.

[60] Kaloyeros AE, Pan Y, Goff J, Arkles B (2020). Review—Silicon nitride and silicon nitride-rich thin film technologies: state-of-the-art processing technologies, properties, and applications. ECS J. Solid State Sci. Technol..

[61] Arx MV, Paul O, Baltes H (2000). Process-dependent thin-film thermal conductivities for thermal CMOS MEMS. J. Microelectromech. Syst..

