# Supplementary material for: Extending the spectrum of fully integrated photonics to submicrometre wavelengths
Source: Nature. 2022 Sep 28;610(7930):54–60. doi: 10.1038/s41586-022-05119-9 (PMC9534754; doi:10.1038/s41586-022-05119-9)
Supplement: Supplementary file 1 — Supplementary Notes 1–4 and Figs. 1–9. [file 41586_2022_5119_MOESM1_ESM.pdf]

nature portfolio

https://doi.org/10.1038/s41586-022-05119-9

Supplementary information

Extending the spectrum of fully integrated photonics to submicrometre wavelengths

In the format provided by the authors and unedited

Supplementary Information for

Extending the spectrum of fully integrated photonics

Minh A. Tran[1,*], Chong Zhang[1,*], Theodore J. Morin[2,*], Lin Chang[2†], Sabyasachi Barik[1], Zhiquan Yuan[3], Woonghee Lee[1], Glenn Kim[1], Aditya Malik[1], Zeyu Zhang[1], Joel Guo[2], Heming Wang[3], Boqiang Shen[3], Lue Wu[3], Kerry Vahala[3], John E. Bowers[2], Hyundai Park[1], Tin Komljenovic[1‡]

[1]Nexus Photonics, Goleta, California 93117, USA.

[2]Department of Electrical and Computer Engineering, University of California, Santa Barbara, CA 93106, USA.

[3]T. J. Watson Laboratory of Applied Physics, California Institute of Technology, Pasadena, CA, USA.

[*]These authors contributed equally

Corresponding authors: [†](linchang@ucsb.edu), [‡](komljenovic@nexusphotonics.com).

Supplementary note I: Heterogeneous III-V/SiN photonic platform fabrication process

The fabrication flow of our platform in this work includes SiN patterning for passive devices, GaAs epi bonding and processing for active devices and finally cladding and metal processes. The entire process takes place at a wafer scale (100mm Si wafer) and is readily scalable to larger wafer size. Detailed descriptions are below:

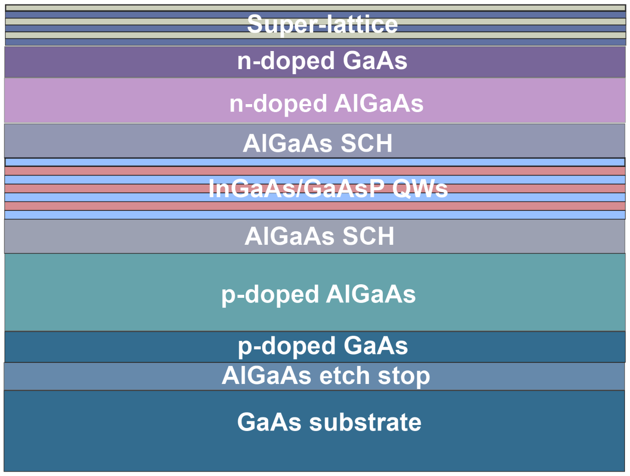

Supplementary Fig. 1: Active component epitaxial structure. The epitaxial structure consists of a super-lattice layer, n-doped GaAs, n-type AlGaAs, AlGaAs separate confinement heterostructure with InGaAs/GaAsP QWs, p-doped AlGaAs, p-doped GaAs and an AlGaAs etch stop layer.

- From a Si bulk substrate wafer, a high-quality silicon dioxide layer was formed on the surface by thermal oxidation to create the bottom optical cladding layer. A stoichiometric SiN layer of 350 nm thickness was then deposited with Low Pressure Chemical Vapor Deposition (LPCVD) to form the SiN waveguide core layer. Subsequently, the SiN layer was patterned with a 248 nm lithography system and a fluorine-based dry etch to form waveguides and passive components. All the alignment marks for further lithography steps were also formed on this layer.

- GaAs coupons were bonded to the SiN using an optimized O_2 plasma activated direct bonding procedure. A nm-scale thin layer of Al_2O_3 (nominally 7 nm) was deposited on the epi surface as an adhesion layer before bonding, and a 12-hour long annealing was carried out at 150°C after bonding to enhance the bonding strength of the two

materials.

- The GaAs substrate was partially removed with mechanical polishing, and substrate removal was completed with a chemical etch, resulting in a 2-μm thick GaAs epitaxially grown stack bonded on top of the patterned SiN.

- Multiple lithography and etching steps were carried out in sequence to form the GaAs active device structures, followed by metallization steps to form P and N electrodes. A wet etch was carried out to remove excess III-V material to preserve the performance of the passive components.

- In the last stage, additional dielectric structures were deposited to form the SiN/GaAs couplers, sidewall passivation and the top optical cladding. Finally, metal depositions and dry etch were carried out to form microheaters, probe pads and electrical connections to the active devices.

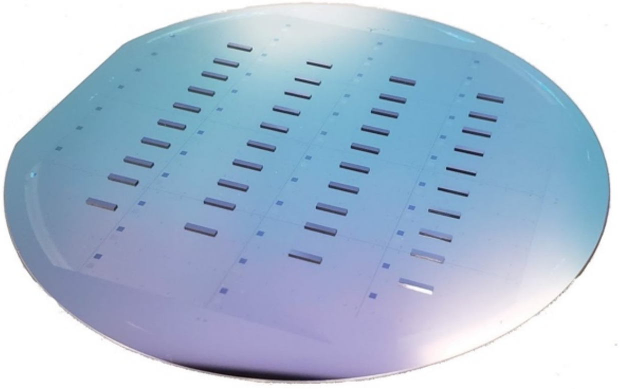

Supplementary Fig. 2: A wafer with multiple epis bonded on and processed at the same time.

In the present work, the lasers, amplifiers, photodetectors and phase modulators all share the same epitaxial structure, shown in Fig. (1). The structure consists of a GaAs p-n junction with strained quantum wells that were optimized for TE mode operation and for a target luminescence of 980 nm. Additional epitaxial layers were introduced to assist in processes related to bonding (e.g. superlattice layers) and substrate removal (an AlGaAs etch stop layer). Also, to accommodate the bonding orientation, the epitaxial growth starts with p and ends with n-type material, the opposite of the conventional layer order on native

substrates. The epitaxy was grown by metal-organic chemical vapor deposition (MOCVD) at a commercial vendor.

All the devices are characterized at 980 nm wavelength except for the modulators that were characterized at an offset wavelength (1060 nm). To unite a variety of operating wavelengths for the future PICs which require multiple wavelength generation and operation, we have also demonstrated the capability of bonding different epis onto the same wafer, as shown in Fig. (2). Future work will report and discuss these results.

Supplementary note II: III-V to Low-index couplers

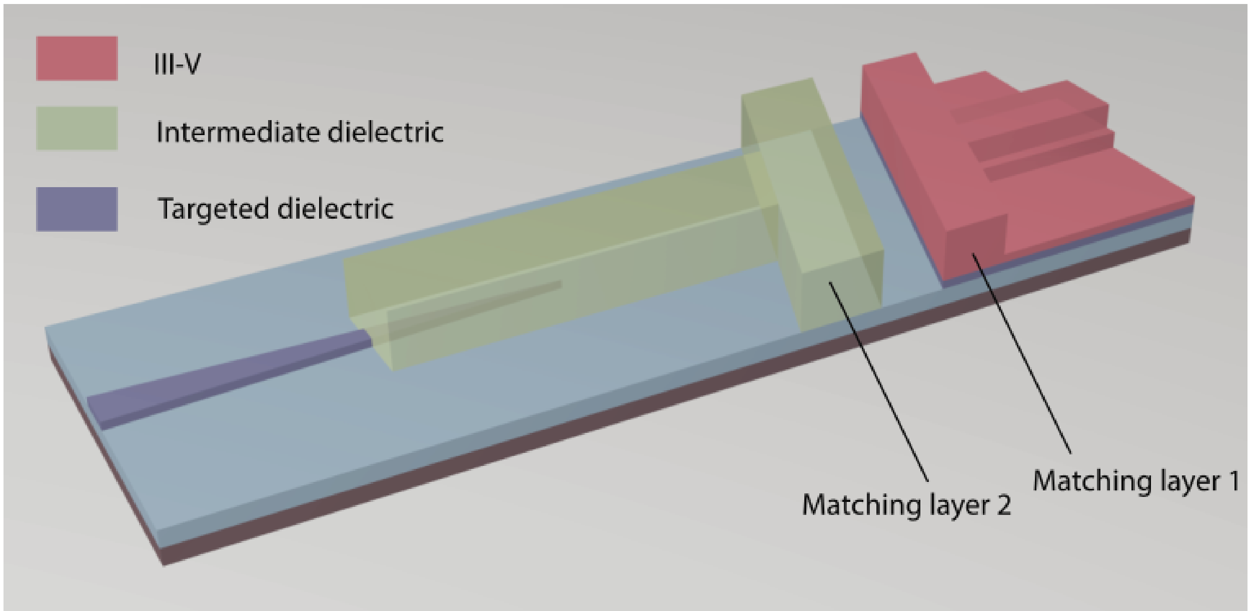

Supplementary Fig. 3: Heterogeneous coupler design. Schematic of the structure to couple III-V and dielectric waveguides.

Compared to previous heterogeneous couplers based on direct tapers between Si and III-V, in this work we use a different coupling strategy to enable efficient mode transition between III-V and dielectric waveguide with large refractive index difference. As shown in Fig. (3), in our structure, the mode transition is achieved via butt coupling. There are several critical points in our approach: first, to match the size of mode between III-V and the targeted dielectric waveguide, an intermediate transition section is used. The thickness of this intermediate dielectric layer is similar to that of the III-V waveguide, so that the mode sizes are matched; second, to eliminate the reflection from the coupler, which is usually

a significant obstacle for butt coupling, we implement one or both of the matching layers (and interface angle) as sketched depending on the device functionality. Finally, in order to transition the mode from intermediate section into the targeted waveguide, the index of the intermediate dielectric material needs to be lower than that of the targeted dielectric material. A taper structure is patterned in at least one of the intermediate or the targeted dielectric layer, depending on the device functionality, to reduce the transition loss.

Supplementary note III: Dual-ring tunable laser

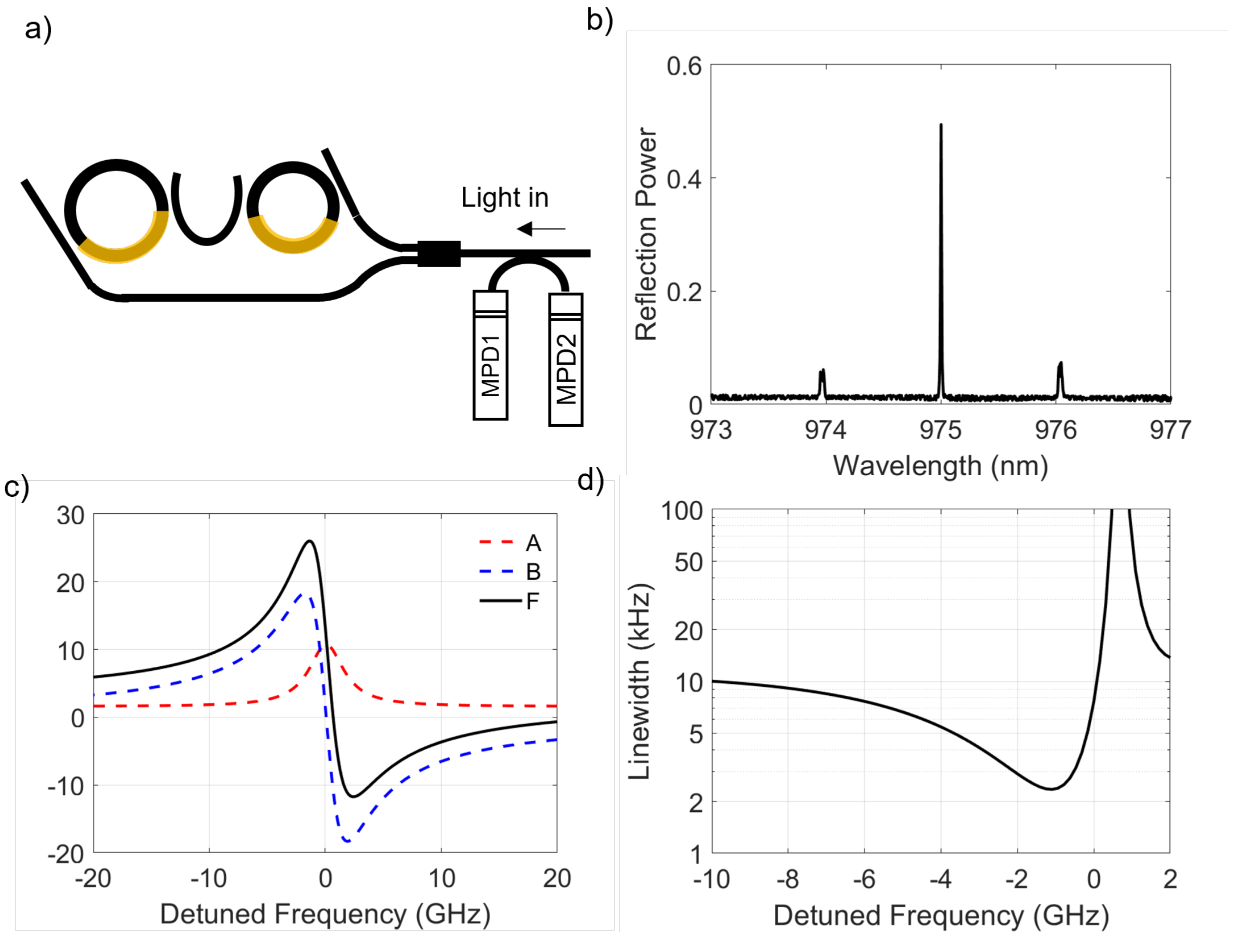

Supplementary Fig. 4: Laser external cavity design a. The schematic of a test structure for characterizing a dual-ring tunable mirror, featuring two monitor photodiodes (MPD). The ring radii are 70 and 72 um. **b.** The measured reflection spectrum from the dual-ring mirror when the two rings were aligned at 975 nm. **c.** Calculated linewidth reduction factors A, B and F. **d.** Calculated laser linewidth as a function of frequency detuning from the Vernier ring's resonance's peak.

The tunable laser described in the main text was designed following [1]. The ring radii were 70 and 72 um, resulting in Vernier free spectral range (FSR) of about 40 nm, which is designed to be roughly two times of the 3 dB gain bandwidth to guarantee stable single mode lasing across the whole tuning range.

The tunable back mirror is characterized by measuring the reflection spectrum from a separate test structure (Fig. 4 a). After the two ring filters are tuned to maximize the photocurrent at the output monitor photodiode (MPD2) at a launched wavelength from an external laser source, the input wavelength of the external laser is swept while measuring photocurrent at input and output monitor photodiodes (MPD1 and 2). Fig. 4 b. shows the reflection spectrum measured by taking the ratio of the photocurrents from MPD1 and 2.

The III-V/SiN heterogeneous laser achieves low noise performance thanks to the ring's resonance feedback through two effects: photon life-time enhancement and the detuned loading effect [1]. As illustrated in Fig. 4 c., the first effect – whose magnitude is represented by factor A – resulted from increased lifetime of photons circulating inside the low loss ring. The second effect – whose magnitude is represented by factor B – resulted from an optical negative feedback loop caused by the carrier plasma effect of the semiconductor's gain materials, occurring when the lasing wavelength is detuned to the red side of the resonance peak. The fundamental (or Lorentzian) linewidth of the laser is reduced by a factor of $F^2 = (1 + A + B)^2$ when connected to its dual-ring mirror extended cavity. Fig. 4 d. plots the anticipated fundamental linewidth of the laser at detuned frequency using the aforementioned equations. We predict a fundamental linewidth ranging from 2-10 kHz depending on the level of frequency detuning, which is in good agreement with our measurements.

Fig. 5 shows a diagram of the phase noise measurement system used in this experiment.

Supplementary note IV: High temperature performance

This section presents additional high temperature performance data: We show LIV characterization, spectra, frequency noise spectra which further illustrate and support the claims of the main text. Fig. 6 shows the high temperature measurement stage used to collect the data used in main text Fig. 4.

Fig. 7 shows LIV measurements on a single FP laser, taken both while increasing its temperature and as its temperature was being lowered back to room temperature, demonstrating that the high temperature operation did not degrade the laser material. In fact, we

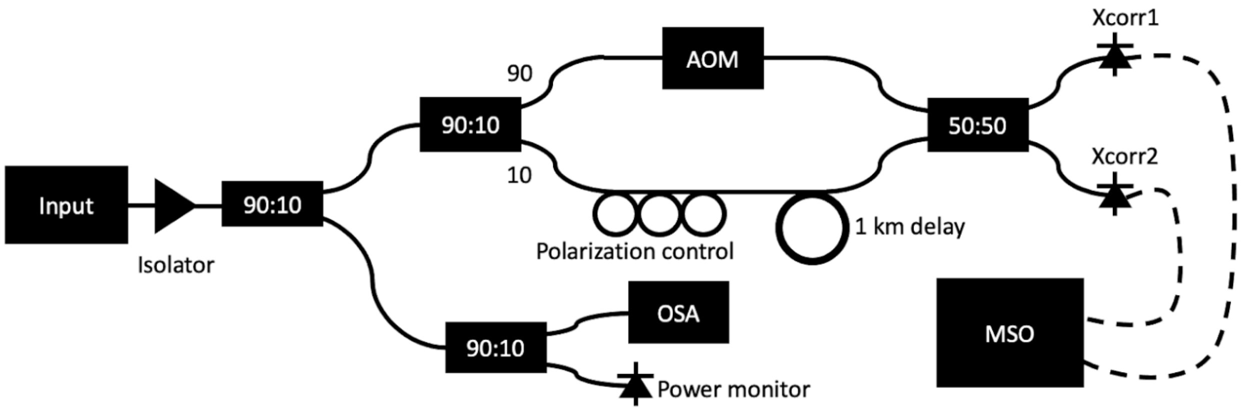

Supplementary Fig. 5: Phase noise measurement schematic. Phase noise was measured as described in the methods section.

observe that the LIV characteristics improved after high temperature operation, which may be explained by some annealing of the electrical contacts. The difference between measurements before and after heating are especially pronounced at low temperatures. We note that trends in our VI data at higher temperatures are obscured by inconsistent probing: Thermal

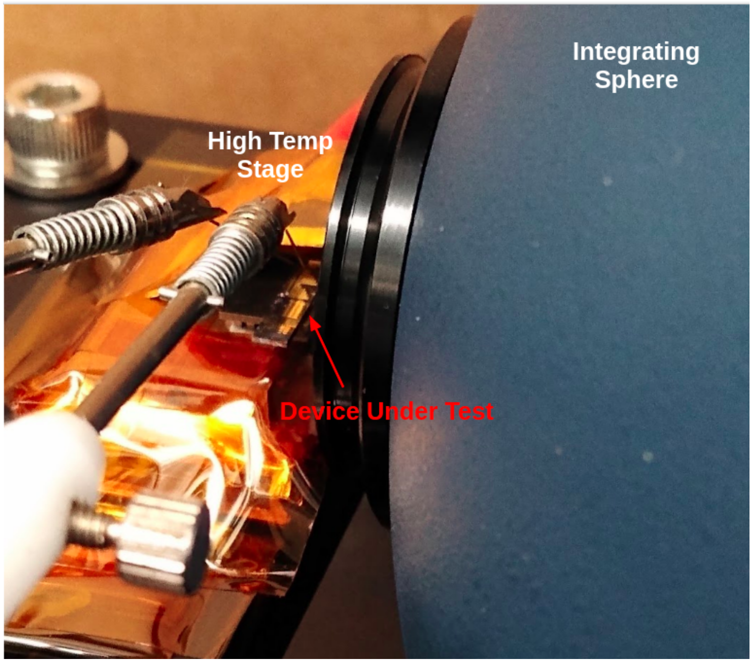

Supplementary Fig. 6: High temperature stage. High temperature measurement stage configured for LIV measurement with Newport integrating sphere. The stage was also used for fiber measurements with the integrating sphere replaced by a lensed fiber.

expansion in the stage required that devices be manually re-probed between measurements at different temperatures, and air currents around the hot stage made the microscope view appear to vibrate.

Fig. 8 shows the complete set of FP spectra whose peak wavelengths are plotted in main text Fig. 4 e. The wavelength versus temperature fit from these spectral plots was used to calibrate the integrating sphere data throughout the paper.

Fig. 9 shows the frequency noise measurements of main text Fig. 3d. The best Lorentzian linewidth measurements were achieved by hand-tuning the battery current sources, including the stage thermo-electric cooler, which was otherwise controlled by a PID loop. Additionally, as with the LIV measurements, devices were re-probed at each temperature despite air currents interfering with the microscope view. As a result, operating parameters did not give consistent results over large time intervals, and low offset noise performance was inconsistent. Nonetheless, linewidth measurements were reproducible over a moderate time interval once suitable operating conditions were found. Considering the visible presence of air currents around the hot stage, the persistence of narrow linewidth performance is espe-

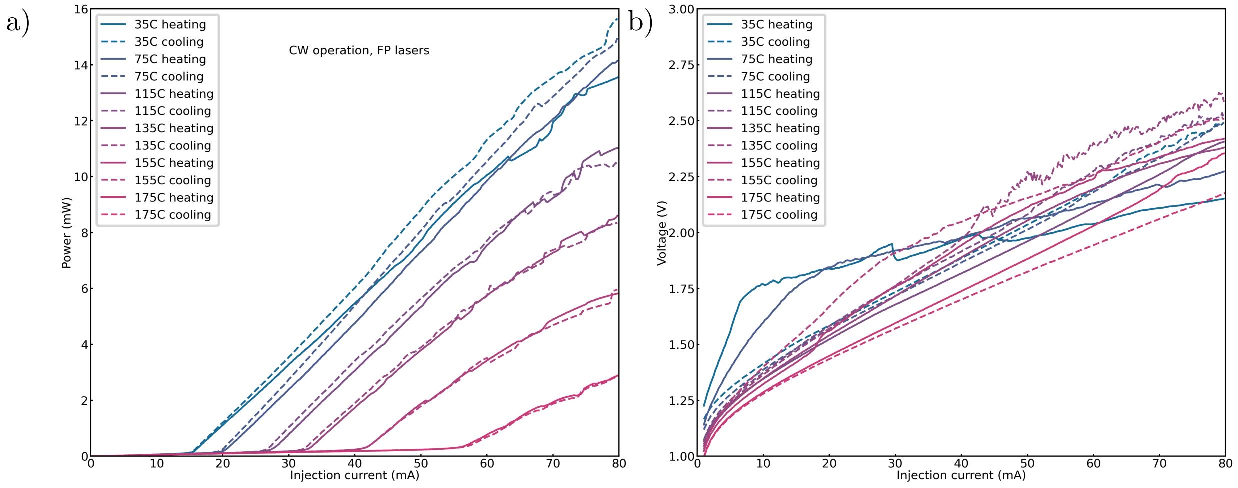

Supplementary Fig. 7: FP laser LIV characteristics. a. Power vs. injected current in an FP laser at various temperatures. **b.** Voltage vs injected current at various temperatures. For both figures, solid lines indicate that the measurement was taken while increasing the stage temperature from room temperature, and dashed indicate that the temperature was being lowered from the maximum measurement temperature at 195C. The measurements taken after heating show slight improvements, possibly due to annealing effects during high temperature operation.

cially impressive, and variation caused by variability of air currents near the device (along with re-probing and re-coupling the presence of air-currents) explains the absence of a clear temperature dependence in the frequency noise spectra. This data is reported despite the lack of a clear trend to transparently support claims of high temperature performance in the main text. A more stable, fully enclosed high temperature measurement environment might reveal temperature dependence trends and deliver superior performance.

The Lorentzian linewidth throughout this paper was determined by smoothing the fre-

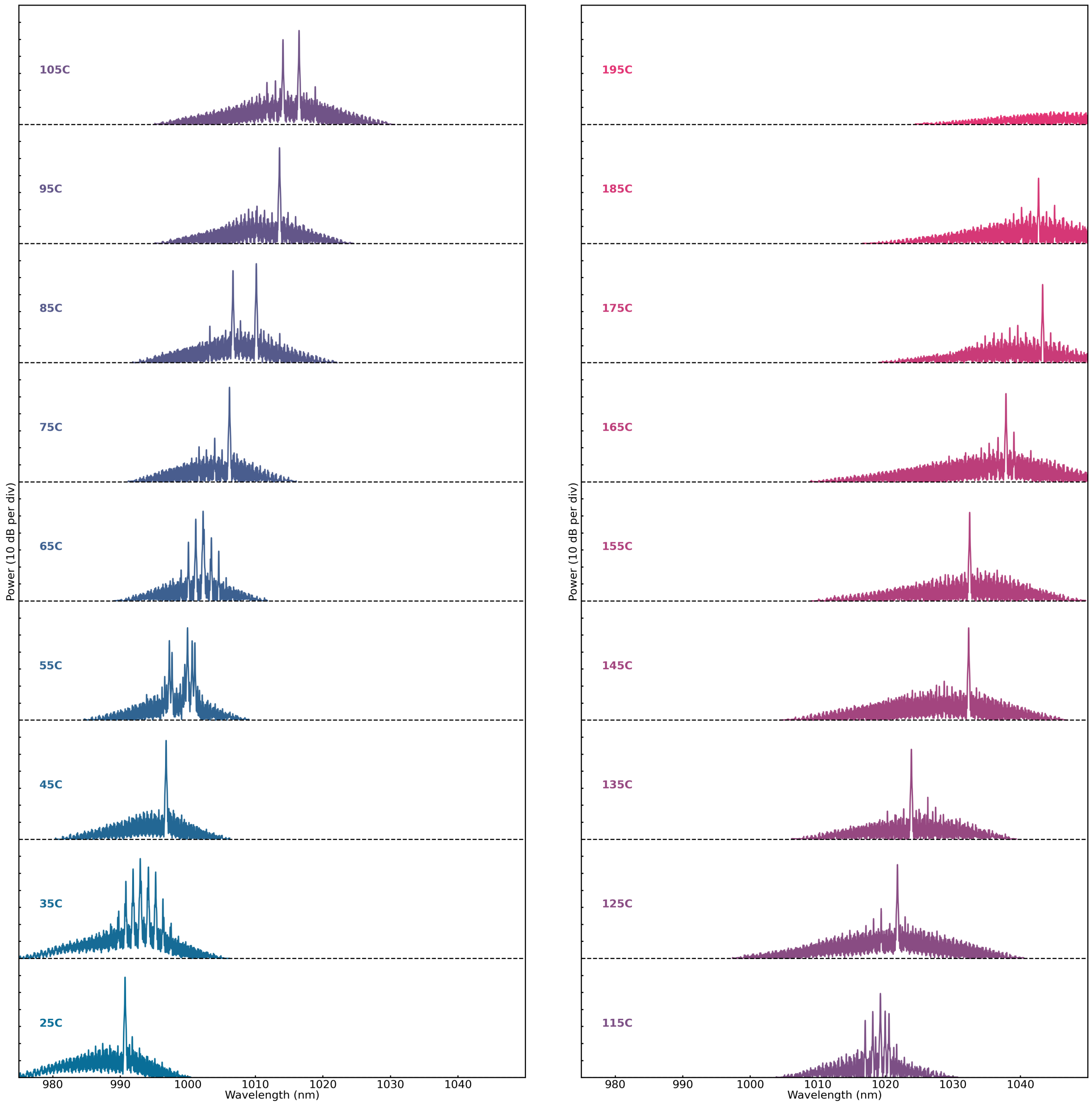

Supplementary Fig. 8: FP laser spectra. FP laser spectra from 25C up to 195C. All measurements were taken at 80mA injection current.

quency noise data, extracting the minimum value and multiplying by 2π. While the measured frequency noise appears to increase at very high frequencies, this trend is due to the limitation of the measurement above half of the self-heterodyne beating frequency.

---

[1] M. A. Tran, D. Huang, and J. E. Bowers, Tutorial on narrow linewidth tunable semiconductor lasers using si/III-v heterogeneous integration, APL photonics **4**, 111101 (2019).

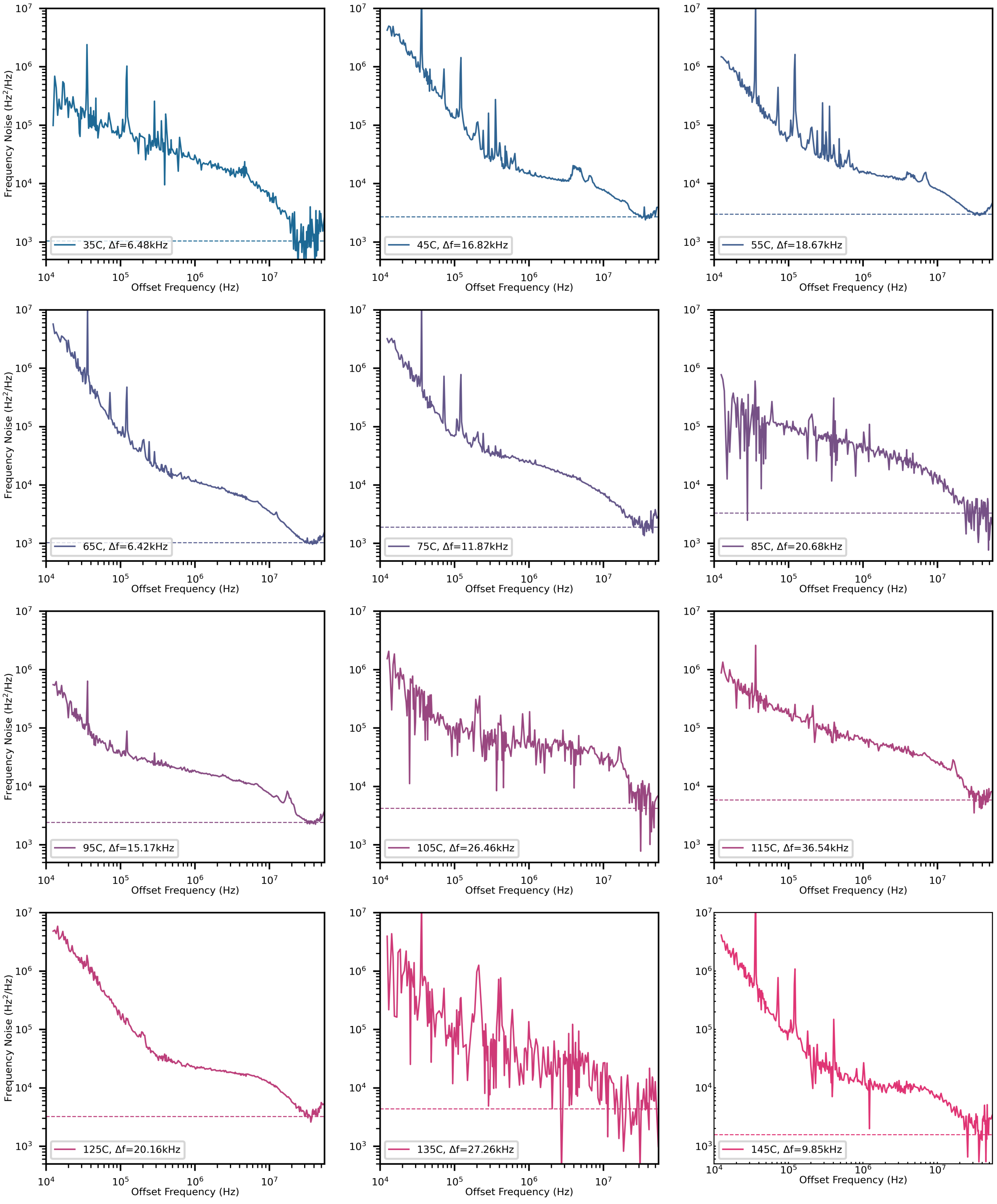

Supplementary Fig. 9: Tunable laser frequency noise. Frequency noise data for a tunable laser operating at various temperatures. Δf (the Lorentzian linewidth) is given by 2π times the white noise floor.
